# A Rare Case of Urachal Cyst in a Patient With Uterine Fibroids

**DOI:** 10.7759/cureus.21092

**Published:** 2022-01-10

**Authors:** Anadi S Tasa, Sandeep Dey, Suren Dutta, Dhrubajit Gogoi, Bikash Bora

**Affiliations:** 1 Surgery, Jorhat Medical College and Hospital, Jorhat, IND; 2 Anaesthesiology, Jorhat Medical College and Hospital, Jorhat, IND; 3 Obstetrics and Gynaecology, Niramoy Hospital and Research Centre, Jorhat, IND; 4 Anaesthesia, Sanjivani Hospital, Jorhat, IND

**Keywords:** urachal cancer, infected urachal cyst, single stage correction, cystectomy, uterine fibroids, urachal anomalies, urachal cyst

## Abstract

A 41-year-old woman presented to the emergency department with pain in her abdomen during menstruation. On examination, we detected a cystic lump in the midline, just below the umbilicus. Ultrasonography of the whole abdomen was suggestive of uterine fibroids with a probable mesenteric cyst. Computed tomography of the abdomen confirmed an otherwise asymptomatic, silent, urachal cyst connected to the umbilicus and urinary bladder by obliterated bands. The cyst was explored and removed surgically under combined spinal-epidural anesthesia, following a single-staged approach. A total abdominal hysterectomy with bilateral salpingo-oophorectomy was subsequently performed. Urachal cysts are rare congenital anomalies. Any unexpected finding on clinical examination should alert clinicians for further evaluation and treatment.

## Introduction

Urachal cysts are rare congenital urachal anomalies that are typically detected in childhood and rarely in adults [1,2]. These cysts affect 1 in 5000 live births [3]. Urachal anomalies are more common among males (male:female = 2:1) [4]. Most cases remain asymptomatic, and patients usually present to the emergency department with acute abdomen [2]. Rarely, the cyst can drain through the bladder or peritoneum, mimicking urinary tract infection or peritonitis [4,5]. Given the varied clinical features and rarity of presentation, a high suspicion index is necessary for the diagnosis.

## Case presentation

A 41-year-old woman (parity two) presented to the emergency room with pain in her abdomen during menstruation for the past seven months. Abdominal pain was gradual in onset, colicky in nature, and appreciable mainly over the lower abdomen. It was moderate to severe in intensity and was relieved with sleep or medication. The pain was associated with heavy menstrual flow, with occasional clots. The patient more frequently experienced the pain during the initial days of menstruation, and it decreased progressively in intensity after that. However, the patient had noted a change in the nature of her pain over the past two months, in that it persisted even after cessation of menstrual flow, affecting her daily activities.

The patient had attained menarche at the age of 13 years, and her periods were otherwise regular with average flow and duration. Her last childbirth via spontaneous vaginal delivery was eight years prior to the current presentation. On examination, the patient was anxious and afebrile. Her vitals (blood pressure, pulse rate, respiratory rate) were stable. No significant findings were noted on inspection. There was no superficial tenderness, guarding, or rigidity. No organomegaly was felt on deep palpation. Mild tenderness was present over the lower part of the abdomen.

A globular lump of ~5 cm in diameter was palpable in the midline below the umbilicus. The margins were well defined, the surface was smooth, and the consistency was smooth to firm. The lump was nontender, nonreducible, noncompressable, nonpulsatile, and did not move with respiration but did demonstrate some side-to-side mobility. The lump tended to be less prominent on the leg-rising test. Hernial sites were intact. On percussion, the typical tympanic note was present over the abdomen, except over the lump, where the note was dull. On auscultation, normal peristaltic sounds were heard over the abdomen, and there was no bruit over the lump. No abnormality was detected on digital rectal examination. The uterus was bulky on vaginal examination.

**Figure 1:**
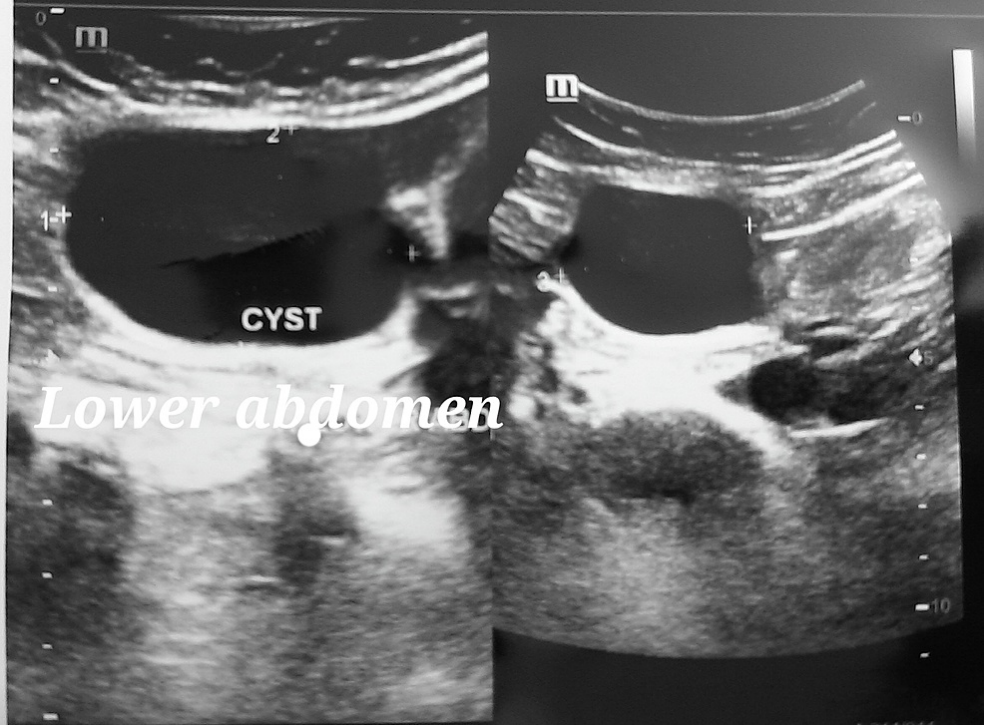
Ultrasonography showing a cystic mass in the lower abdomen

Ultrasonography (USG) of the entire abdomen was advised and was suggestive of a mild bulky retroverted uterus with intramural fibroids and a well-defined anechoic cyst (40 × 33 × 30 mm) in the hypogastrium, with no solid component or calcification, and abutting the peritoneal surface of the anterior abdominal wall, which suggested a probable mesenteric cyst (Figure 1).

**Figure 2:**
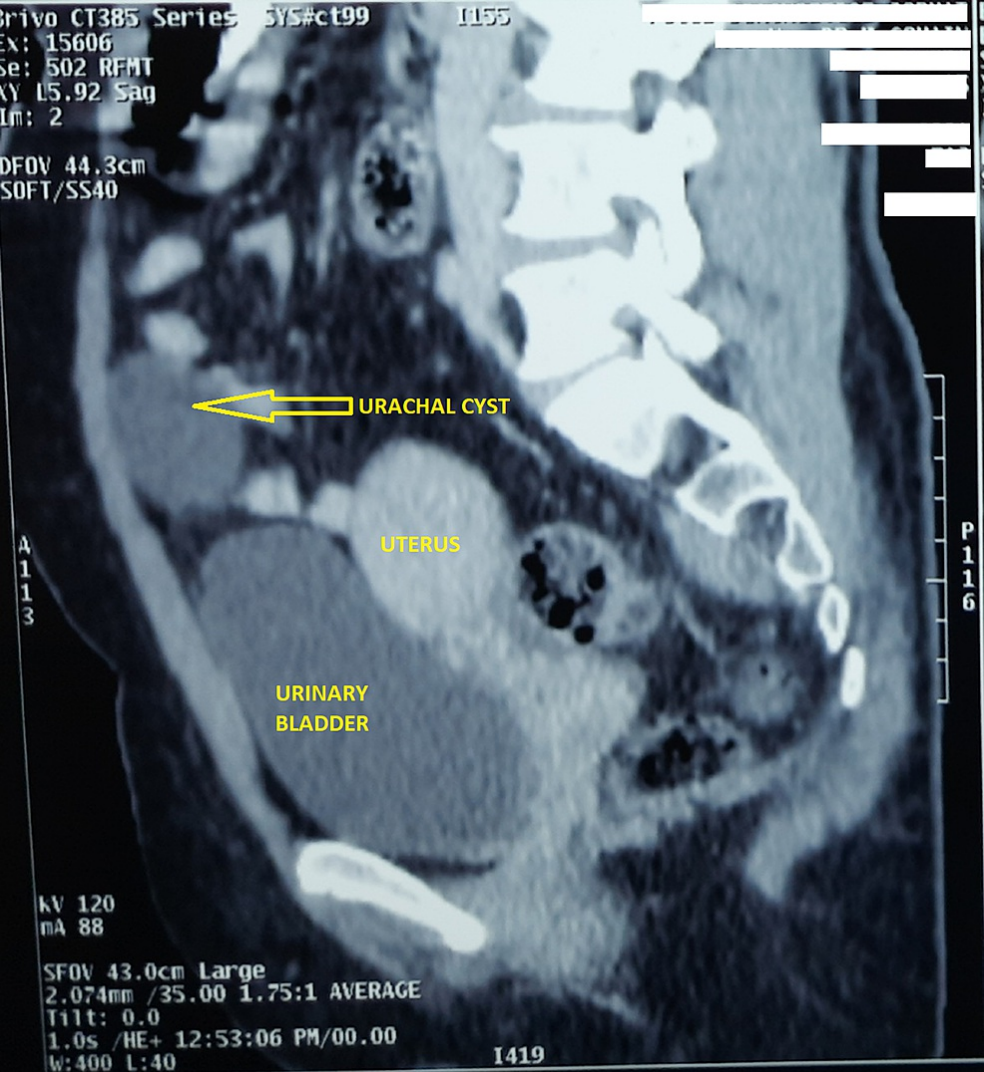
CT scan abdomen showing urachal cyst Abbreviation: CT, computed tomography.

Further evaluation of the cyst via computed tomography (CT) of the abdomen showed a well-defined, thin-walled, hypodense, intraabdominal cystic lesion just below (approximately 2 cm) the level of the umbilicus and along the course of the urachus, measuring approximately 65 × 37 × 43 mm (horizontal × anterior-posterior × transverse). No calcification or septation was noted. A thin, nonenhancing band was seen extending cranially from the upper pole of the cyst to the umbilicus and an obliterated urachal band from the lower pole of the cyst to the apex of the urinary bladder, suggestive of a urachal cyst (Figure 2).

Other routine investigations, including chest radiography and electrocardiography, were within normal limits. As the symptoms of dysmenorrhagia persisted and there was a change in the nature of the pain, even with medical management, surgical intervention with total abdominal hysterectomy with bilateral salpingo-oophorectomy and cystectomy were planned. No significant findings were noted during the preanesthetic checkup. Her exercise tolerance was good (METS score 8) and her functional classification was American Society of Anesthesiologists II. She was of average build and height for her race, age, and sex with a normal spine and back. Consistent with the surgical intervention and to continue analgesia in the postoperative period, combined spinal-epidural anesthesia was planned. Antibiotic coverage and mild anxiolytic were started from the night prior to the proposed day of surgery.

**Figure 3:**
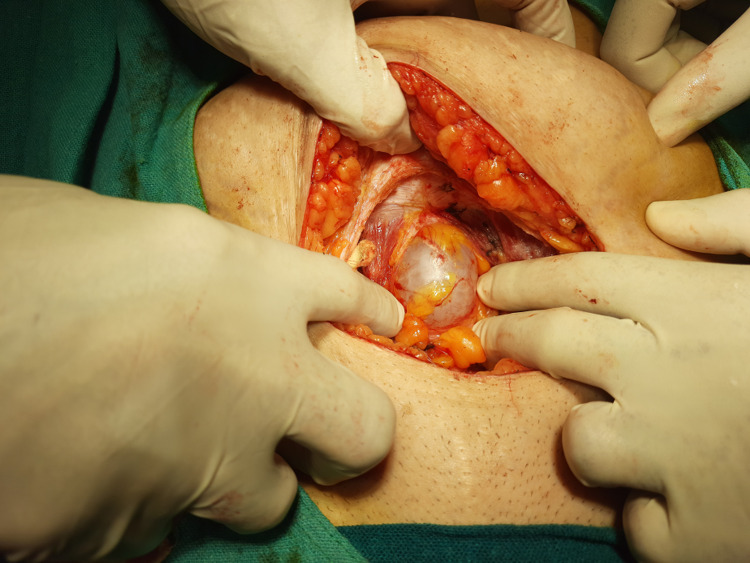
Urachal cyst lying just below the umbilicus

**Figure 4:**
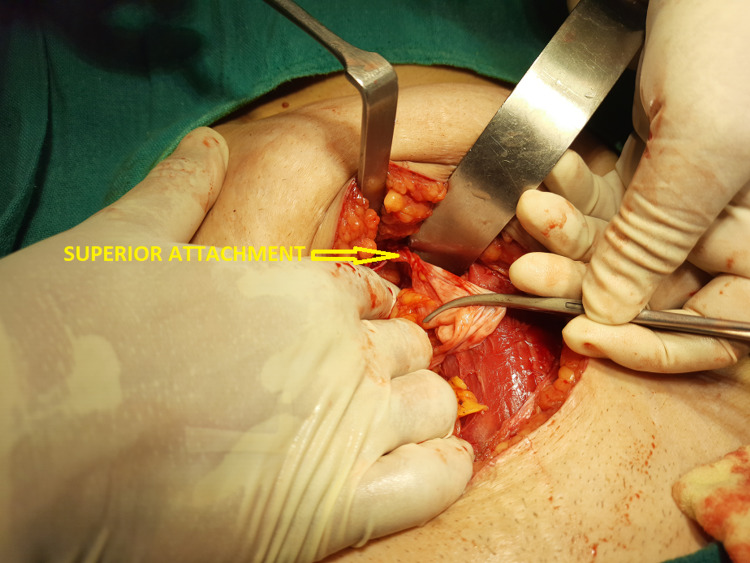
Superior attachment of urachal cyst with the umbilicus

**Figure 5:**
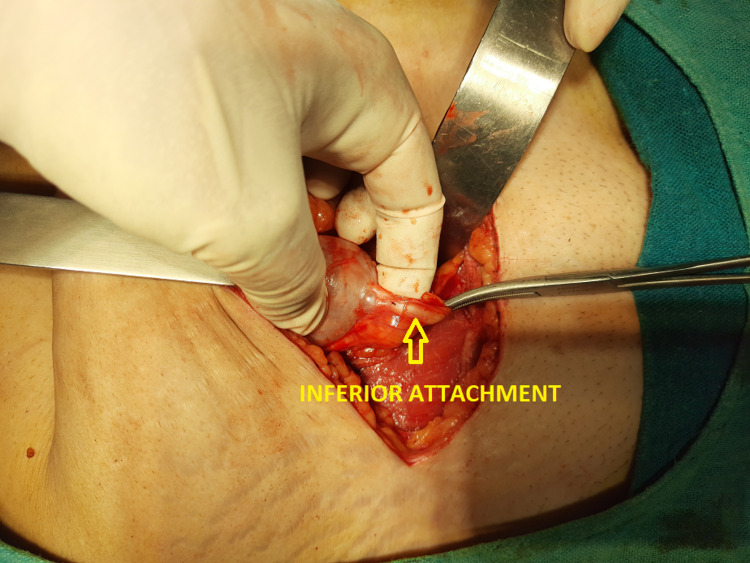
Inferior attachment of the urachal cyst with the urinary bladder

On the day of surgery, after setting up the epidural catheter at the level of L3-L4, a subarachnoid block was administered at L4-L5 using 16 mg bupivacaine heavy 0.5% and 25 μg fentanyl. We approached through a lower midline incision and entered the peritoneal cavity. A globular cystic swelling ~4 cm diameter was noted in the midline, just below the umbilicus (Figure 3). It was attached to the umbilicus superiorly by a cordlike extension (Figure 4). Inferiorly, it was attached to the urinary bladder by a similar band, which pulled up the urinary bladder (Figure 5). The entire urachal cyst was surrounded by dense adhesion and bowel loops. We performed blunt dissection with meticulous hemostasis to dissect the entire urachal cyst. We then approached superiorly and excised the cordlike extension of the urachal cyst with the umbilicus. Subsequently, proceeding inferiorly, we excised the connection of the urachal cyst with the urinary bladder, avoiding injury to the urinary bladder wall. Total abdominal hysterectomy with bilateral salphingo-oophorectomy followed. The intraoperative period was uneventful. Analgesia was maintained postoperatively using epidural top-up of 8 - 10 ml with levobupivacaine 0.0125%. The patient was discharged on postoperative day three, and she was doing well during her checkup two weeks later.

**Figure 6:**
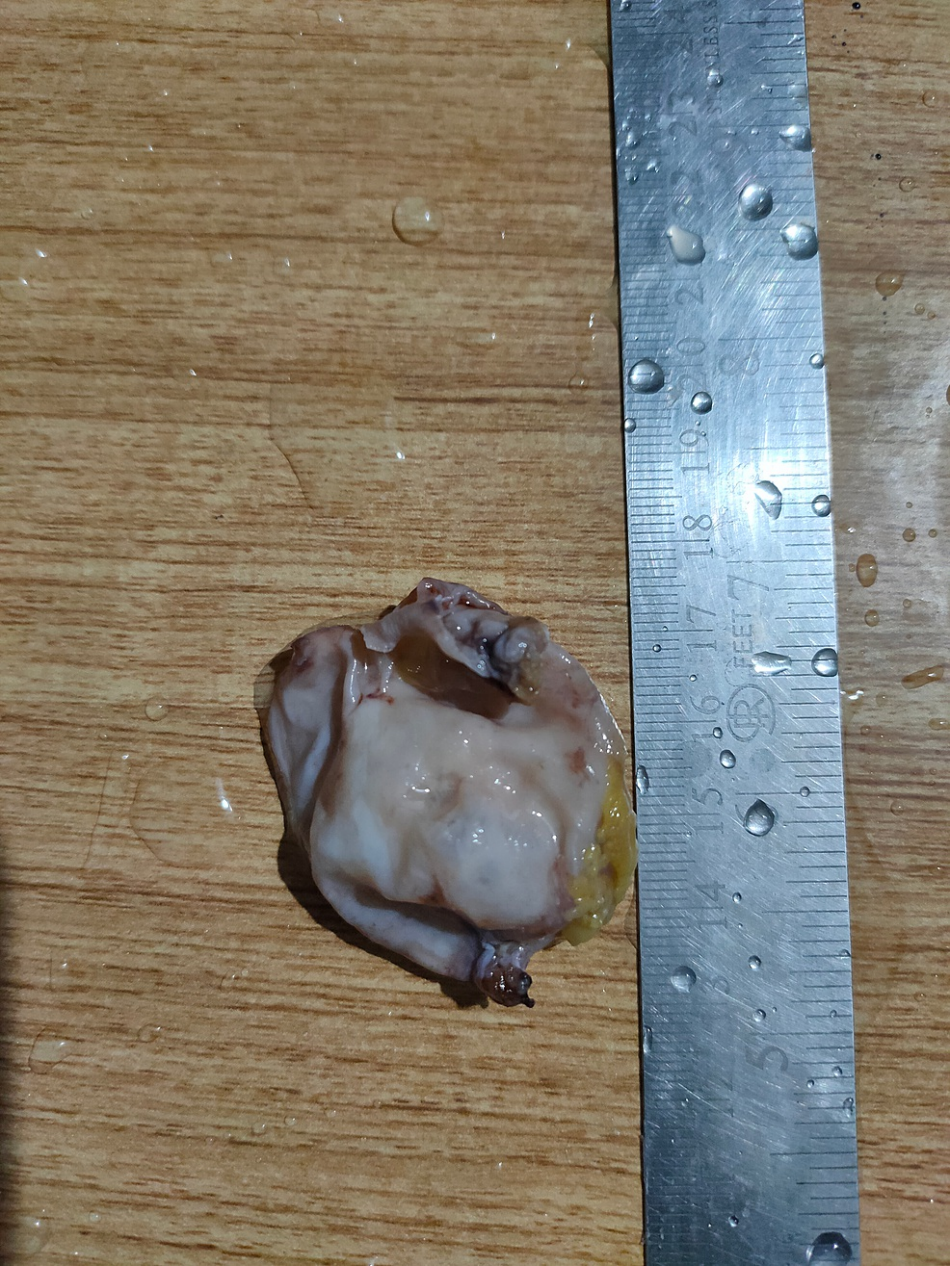
Gross morphology of the urachal cyst

**Figure 7:**
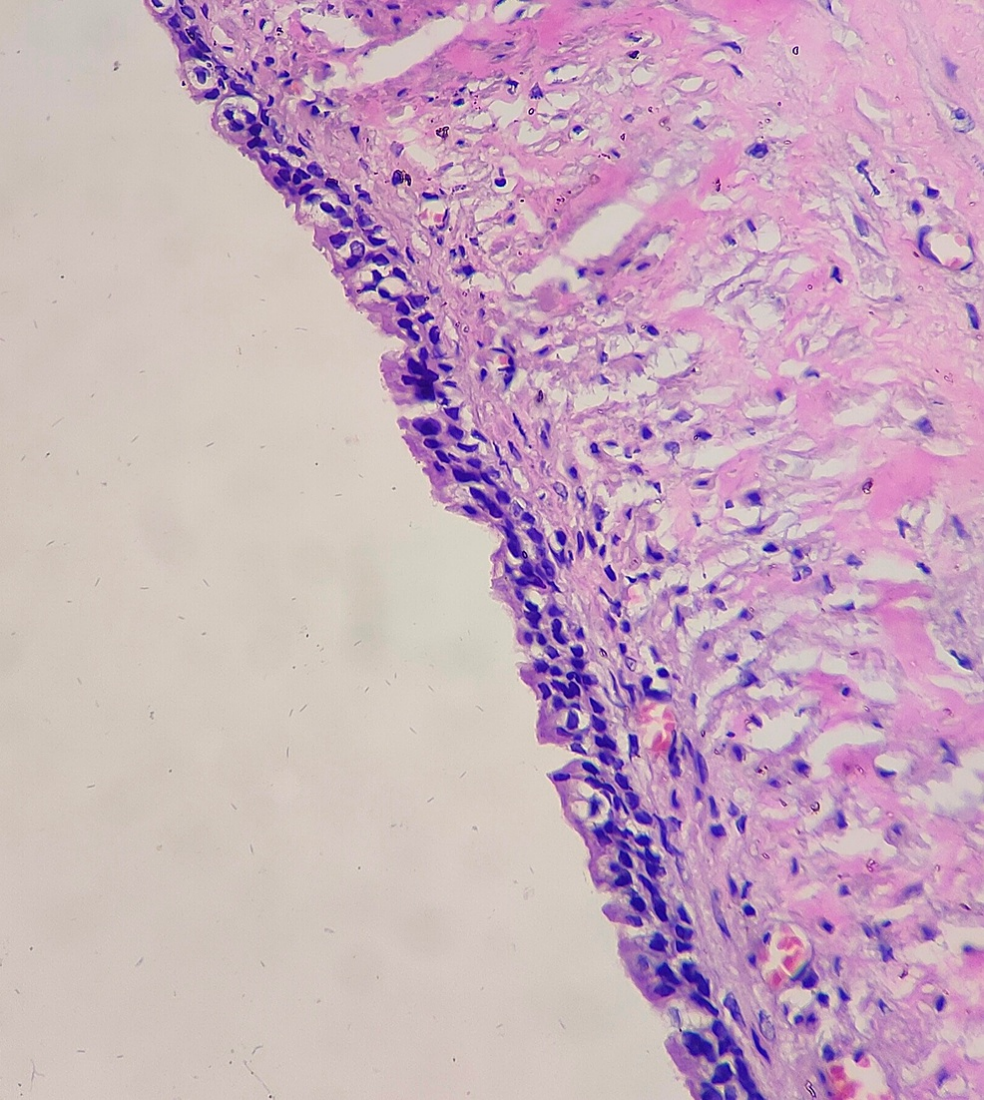
Microscopic view of the urachal cyst

On histopathological examination, the cyst was approximately 4 cm in diameter and had a smooth outer surface with thin walls, and was uniloculated (Figure 6). Microscopically, the cyst had a fibrous wall lined by single to multiple layers of the attenuated epithelium with no cellular atypia, suggestive of a benign urachal cyst. The cystic fluid was clear, without any turbidity, and there was no growth after overnight incubation (Figure 7).

## Discussion

The urachal cyst arises from an unobliterated urachus (or medial umbilical ligament), which itself is the vestigial remnant of the cloaca and the allantois [6]. In early fetal life, the urachus extends as a tubular structure from the apex of the urinary bladder to the umbilicus. It begins its involution by late embryonic life, via fibrous proliferation, persisting until before birth (by the fifth to the seventh month of gestation) only as a thin fibrous band that lies between the fascia transversus and parietal peritoneum [7]. Any defect in its natural course of involution gives rise to urachal anomalies, which can be of four types: patent urachus (~50%), umbilical-urachal sinus (~15%), vesicourachal diverticulum (3-5%), and urachal cyst (~30%) [8].

The urachal cyst ensues when the urachus is involuted at either endpoint but persists in between. Most commonly, it is found around the lower one-third of the urachus [9]. Urachal cysts are usually asymptomatic and are typically detected when they become symptomatic as they enlarge or during some other routine radiological investigation [10]. The most common complication of a urachal cyst is an infection, with the most typical isolates of an infected cyst being Escherichia coli, Enterococcus faecium, and Klebsiella pneumonia [11,12]. When infected, an otherwise asymptomatic patient may present with symptoms of acute abdomen mimicking obstructed hernia, acute appendicitis, Meckel's diverticulitis, or pelvic inflammatory disease [13]. An infected cyst rarely ruptures spontaneously, presenting as peritonitis [5]. Although rare, malignant transformation is a reported complication of urachal anomaly [14,15]. Hence, whenever detected, total removal of the cyst is the treatment of choice, given the risk of reinfection (30%) and malignant transformation [4,8].

Radiologic investigations play a pivotal role in differentiating a urachal cyst from other causes of acute abdomen. A simple cyst appears as a fluid-filled cavity on a CT scan or USG abdomen [16-18]. When infected, there are features of increased attenuation on CT scans or mixed echogenicity on USG [16,18]. Malignant cysts may produce psammoma calcification on CT scans [4,19] or high echogenic calcifications with solid components on USG [20]. Although CT scans help to better delineate the USG findings, both modalities are less sensitive for differentiating an infected urachal cyst from a malignant one, and histological diagnosis is thus sometimes warranted before any definitive surgical intervention is attempted [9,11,16,21,22].

For a urachal cyst, complete surgical excision of the cyst is the treatment of choice. Two methods have been described in the literature: a single-stage excision and a two-stage approach, whereby the initial incision and drainage of the cyst is followed by secondary excision. Many authors have reported that the two-stage approach is associated with reduced hospital stay and a decreased complication rate [23-25]. In our case, which was an unexpected diagnosis and did not have any other features of infective etiology, we opted for a single-stage approach, with no reported complications and a healthy follow-up.

## Conclusions

Urachal cysts are rare congenital urachal anomalies that are typically asymptomatic and diagnosed when they produce symptoms secondary to infection or hemorrhage or during some other routine examination. As such, cysts may lie anywhere between the umbilicus and the urinary bladder apex, and they can have varied symptoms, at times mimicking acute abdomen. Rarely, the cyst may rupture, leading to localized or generalized peritonitis. Because timely intervention is the key for any healthy patient outcome, any unexplained mass on clinical examination should alert the clinician to make an alternative diagnosis with relevant clinical and radiologic evaluation.
